# INVASIVE SPECIES: Do Noxious Neighbors Spread Disease?

**DOI:** 10.1289/ehp.118-a524

**Published:** 2010-12

**Authors:** Bob Weinhold

**Affiliations:** **Bob Weinhold**, MA, has covered environmental health issues for numerous outlets since 1996. He is a member of the Society of Environmental Journalists

Invasive plants are known for disrupting the ecologic balance in plant and animal communities.^1^ They also may play a role in the spread of human diseases, according to a study of ehrlichiosis and its relationship to the noxious weed Amur honeysuckle (*Lonicera maackii*).^2^

Ehrlichiosis is an emerging disease that occurs in people and other animals. In people, one of the most prevalent culprits is the bacterium *Ehrlichia chaffeensis*, which causes a form of the disease known as human monocytic ehrlichiosis (HME).^3^ There also have been a few documented cases of HME caused by *Ehrlichia ewingii*. Both bacteria are transmitted by the lone star tick (*Amblyomma americanum*), a vector thought to feed primarily on the white-tailed deer (*Odocoileus virginianus*).^4^

HME was first reported in 1987, and the number of reported cases has risen steadily from about 100 in 1999 to 957 in 2008.^5^,^6^ The 10-fold increase likely is due to a combination of increased incidence, better reporting, and possibly increased exposure to lone star tick habitat through outdoor work and recreation, says Erik Hofmeister, veterinary medical officer with the U.S. Geological Survey (USGS) National Wildlife Health Center.

Amur honeysuckle, first introduced into the United States and Canada from eastern Asia in the 1800s,^7^ was widely used for landscaping, soil erosion control, and wildlife habitat enhancement, but its tendency to invade native settings was noticed as early as the 1920s.^8^ It seldom is used any more, says Robert Schutzki, an associate professor of horticulture at Michigan State University. Nonetheless, it’s well established, often in urban and urban fringe areas, throughout much of the eastern half of the United States and Ontario, Canada.^9^

Noticing the overlapping geographic distribution of HME, lone star ticks, their hosts, and Amur honeysuckle, Brian Allan, now an assistant professor of entomology at the University of Illinois at Urbana–Champaign, set out to assess the relationship among these four factors. With his colleagues he assessed nine natural areas in the St. Louis, Missouri, region, pairing honeysuckle-invaded and uninvaded plots measuring at least 30 m^2^. They also compared invaded plots against those where they removed honeysuckle (either whole plants or just the fruit).

In both situations, they found a strong link between *Ehrlichia*-infected lone star ticks, deer, and Amur honeysuckle. Elaborating on figures published in his paper, Allan says the density of *E. chaffeensis*-infected tick nymphs^10^ in the honeysuckle stands was 25 times higher than in nearby stands of native vegetation, and deer density was 4 times higher. In areas where honeysuckle was removed, he says the density of *E. ewingii*-infected tick nymphs was 17 times lower than in nearby stands of honeysuckle vegetation, and deer density was 5 times lower.

One other invasive plant (Japanese barberry, *Berberis thunbergii*), has been linked with an emerging human illness (Lyme disease).^11^,^12^ Preliminary evidence indicates another invasive plant, garlic mustard, also may play a role in Lyme disease, says Felicia Keesing, an associate professor of biology at Bard College. Allan says the budding evidence suggests additional research on links between invasive plants and human diseases is needed.

Hofmeister is impressed with this study, including the in-depth analysis of the ecology of the disease, and he thinks it can have immediate applications. For instance, he says, “Homeowners could potentially reduce their risk of ehrlichiosis if they cleared the honeysuckle from around their property.”

But Tom Stohlgren, a research ecologist at the USGS’s Fort Collins Science Center in Colorado, is skeptical of the study’s importance, though not entirely negative. “I think this is a tangential direction,” he says. “It’s better to go after the disease source itself, such as long-term increases in deer populations due to predator control, and increased urbanization into deer habitat. This study carries the argument deeper than it may need to go. But it’s an interesting and important link, and I don’t want to lose it.”

## Figures and Tables

**Figure:**
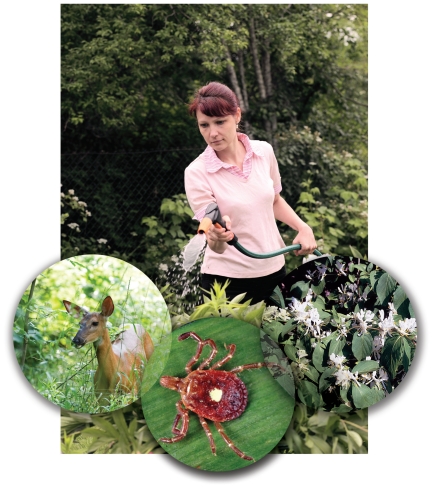
Vectors, hosts, invasive species, and people all living in close proximity may be a recipe for disease.
